# Highly Effective Covalently Crosslinked Composite Alginate Cryogels for Cationic Dye Removal

**DOI:** 10.3390/gels7040178

**Published:** 2021-10-22

**Authors:** Serap Sezen, Vijay Kumar Thakur, Mehmet Murat Ozmen

**Affiliations:** 1 Department of Bioengineering, Yildiz Technical University, Istanbul 34210, Turkey; seraph@sabanciuniv.edu; 2 Biorefining and Advanced Materials Research Centre, SRUC, Edinburgh EH9 3JG, UK; 3 Department of Mechanical Engineering, School of Engineering, Shiv Nadar University, Greater Noida 201314, Uttar Pradesh, India; 4 School of Engineering, University of Petroleum & Energy Studies (UPES), Dehradun 248007, Uttarakhand, India

**Keywords:** alginate, carbodiimide, cryogel, composite, methylene blue, montmorillonite

## Abstract

Currently, macroporous hydrogels have been receiving attention in wastewater treatment due to their unique structures. As a natural polymer, alginate is used to remove cationic dyes due to its sustainable features such as abundance, low cost, processability, and being environmentally friendly. Herein, alginate/montmorillonite composite macroporous hydrogels (cryogels) with high porosity, mechanical elasticity, and high adsorption yield for methylene blue (MB) were generated by the one-step cryogelation technique. These cryogels were synthesized by adding montmorillonite into gel precursor, followed by chemical cross-linking employing carbodiimide chemistry in a frozen state. The as-prepared adsorbents were analyzed by FT-IR, SEM, gel fraction, swelling, uniaxial compression, and MB adsorption tests. The results indicated that alginate/montmorillonite cryogels exhibited high gelation yield (up to 80%), colossal water uptake capacity, elasticity, and effective dye adsorption capacity (93.7%). Maximum adsorption capacity against MB was 559.94 mg g^−1^ by linear regression of Langmuir model onto experimental data. The Pseudo-Second-Order model was fitted better onto kinetic data compared to the Pseudo-First-Order model. Improved porosity and mechanical elasticity yielding enhanced dye removal capacity make them highly potential alternative adsorbents compared to available alginate/montmorillonite materials for MB removal.

## 1. Introduction

**Figure 1:**
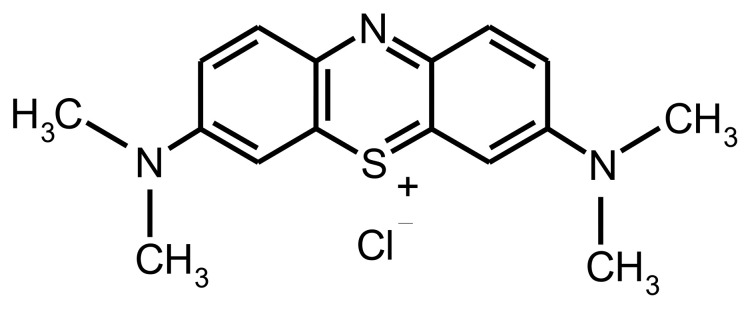
Chemical structure of methylene blue [9].

In recent years, dye contamination in-ground and freshwaters have been a challenging problem due to their non-biodegradability and toxicity to ecosystems. These effluents are released by industrial applications such as textiles, cosmetics, and plastics [1,2]. Although various synthetic dyes accumulated in nature are toxic, cationic dyes such as methylene blue (MB) (Figure 1) pose more damage to the environment and humans due to their charge [2,3]. Therefore, these unfavorable features of dyes have prompted the development of strategies like photodecomposition, coagulation, flocculation, ion exchange, applying chemical oxidizing agents, and adsorption to remove them from contaminated waters [4,5,6]. Among all methods, adsorption has been found to be superior in economical and effective ways for the recovery of synthetic dyes owing to the selectivity of adsorbent types, sorbent reusability, and ease of operation [2,4,7,8].

Various materials such as activated carbon, solid agricultural by-products, carbon-based compounds, metallic compounds, polymers, industrial wastes, and clays are applied for dye adsorption in the forms of porous matrices, nanoparticles, membranes, and bulk materials hydrogels [1,5,10]. Hydrogels have been of interest among these materials due to their promising potential in overcoming various ecological, biological, and industrial problems [11,12]. Specifically, hydrogels have been used to remove dyes in polluted water due to the availability of functional groups for dye attachment, ease of production, processing and handling with a high recovery rate. As a well-known natural polymer-based hydrogel, alginate hydrogel with the advantages of ionic functional groups is commonly utilized to capture ionic dyes [13]. Moreover, alginate hydrogels have been utilized in cationic dye adsorption due to their abundance, being eco-friendly, and low-cost alginate [14]. Alginate is a polysaccharide composed of β-D-Mannuronic Acid (M) and α-L-Guluronic Acid (G) units that possess negatively charged carboxylate groups. Therefore, in divalent cations, alginate forms hydrogel beads by ionic cross-linking [13]. However, these fast ionic cross-linking limits control over the properties of alginate hydrogels, such as porosity and mechanical elasticity due to fast reaction between ionic groups. Generally, the obtained hydrogels possess fast-crosslinked and weak-crosslinked zones that undergo degradation under physiological conditions [15,16,17]. To eliminate these problems in alginate hydrogels, a covalent cross-linking strategy can be applied.

Recently, cross-linking reaction at low temperature under cooling has been a suggested technique to prepare effective macroporous hydrogels, so-called cryogels. Cryogels are prepared at temperatures below the freezing point of the gelation solvent, where solvent crystals are formed in the semi-frozen system. Subsequently, the reaction system is thawed, leaving micrometer sized large pores [18,19]. Although cryogels have superior swelling and mechanical properties, still a need arises to improve their performance. Incorporating nanomaterials like clay into the cryogel structure can form nanocomposite cryogels with enhanced elasticity, bio-functionality, swelling, antibacterial, and adsorption properties [20,21,22].

This study prepared alginate/montmorillonite cryogel adsorbent for MB removal by adding montmorillonite clay into polymeric gel precursors of alginate, followed by covalent cross-linking at low temperatures. Crosslinking reaction was achieved with carbodiimide chemistry whereby 1-Ethyl-3-(3-dimethylaminopropyl) carbodiimide (EDC) and N-Hydroxysuccinimide (NHS) were used to activate carboxylate groups of alginate, later cross-linked with cystamine [23,24]. Various alginate materials including alginate cryo-beads for biological applications [25,26,27], alginate-gelatin cryogels [28], alginate-agarose cryogels and cryo-beads for bioengineering applications [24,29,30,31], methacrylated alginate cryogels for biomedical applications [32,33,34], click alginate cryogel for protein delivery [35], covalently cross-linked alginate cryogels for neural tissue engineering by Mooney et al. [36], and alginate quasi-cryogel beads for dye removal by Erim et al. [7,37] were prepared in the form of beads by ionic cross-linking. For instance, alginate/montmorillonite cryogel-like structures were obtained through ionic cross-linking that were designated as quasi-cryogels [7,37]. However, these materials do not exhibit genuine cryogel features as they lack open macroporosity and toughness. Herein, we obtained alginate cryogels in the cylindrical form by chemical cross-linking utilizing EDC/NHS and cystamine crosslinker that could potentially boost the dye removal capacity. A one-step cryogelation technique was applied to generate these novel true alginate/montmorillonite cryogels, and the resulting materials achieved superior dye adsorption capacity compared to available alginate/montmorillonite adsorbents.

## 2. Results and Discussion

**Figure 2:**
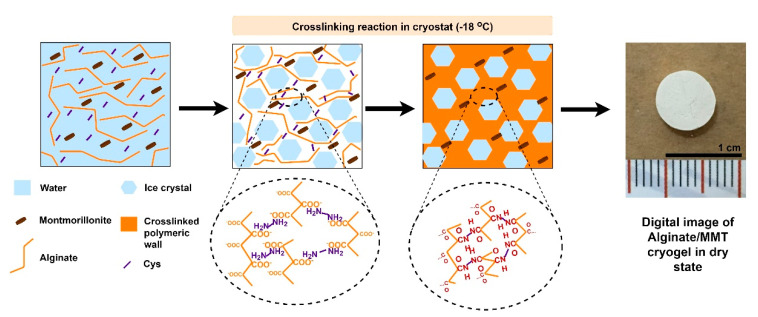
Schematic representation of cryogelation reaction for alginate/MMT macroporous gels and digital image of the dry gel.

Alginate/MMT composite macroporous gels were prepared using a one-step cryogelation method that comprises the preparation of polymeric gel precursor incorporated with MMT clay followed by covalent cross-linking in a moderately frozen state illustrated by Figure 2. Since alginate is a copolymeric polysaccharide owing to carboxylate groups, alginate gels can be formed by amine di-terminated crosslinkers, such as Cys. Therefore, EDC-NHS coupling was also involved in the activation of carboxylate groups, whereby MES was also employed to stabilize the pH of the medium [23,24]. After adding all components into the reaction solution, the mixtures were transferred to cryostat (−18 °C) and ice crystal formation during freezing expelled polymer, crosslinker, MMT, and activator. Therefore, cross-linking achieved in the unfrozen phase and later thawing ice crystals revealed macroporous gel structure.

### 2.1. Characterization of Alginate/MMT Cryogels

**Figure 3:**
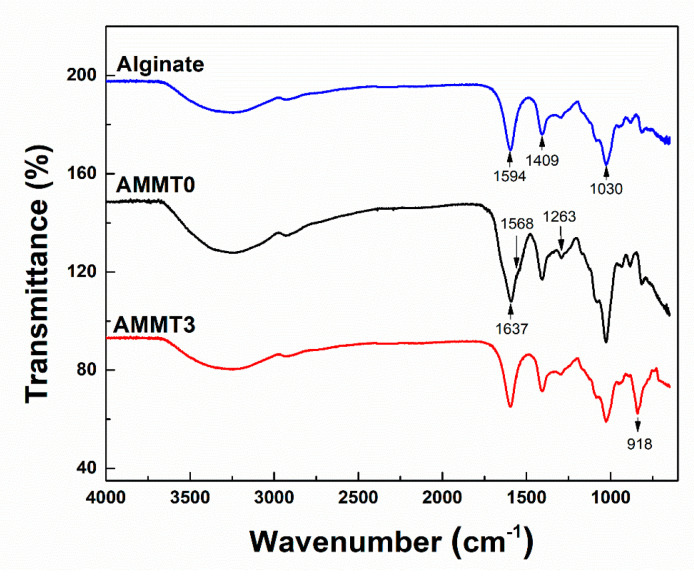
FT-IR spectrum of alginate, AMMT0, and AMMT3 samples. Crosslinking reaction and MMT incorporation were provided by alteration in bands of each specimen.

To investigate the covalent cross-linking reaction between alginate chains and MMT deposition, FT-IR analysis was performed and shown in Figure 3. The spectrum of alginate exhibited broad peaks between 3400 and 3200 cm^−1^, referring to O-H groups. C-O-O groups were identified as C-O stretching at 1409 cm^−1^ [38]. The peak represented at 1030 cm^−1^ shows O-C-O stretching typical for polysaccharides. The spectra of AMMT0 represents a sharp peak at 1637 cm^−1^, which corresponds to the amide I bond. Peaks at 1263 and 1568 cm^−1^ show N-H bending of amide II bonds. Amide I and II bonds formation indicates amidation of the carboxylic acid of alginate, meaning that cross-linking of alginate was successfully achieved by Cys [23]. AMMT3 exhibits peaks around 918 cm^−1^ due to Al-Al-O-H stretching of MMT [39]. Therefore, MMT incorporation into alginate cryogels has also been accomplished.

**Figure 4:**
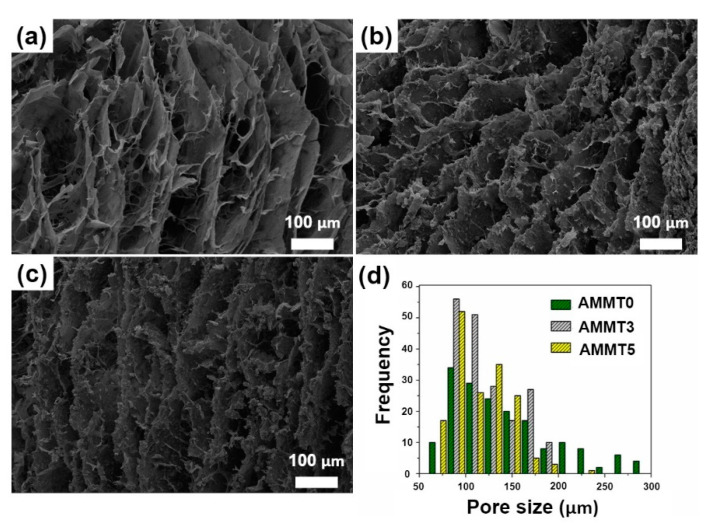
Cross-sectional SEM images and pore size distribution of (**a**) AMMT0, (**b**) AMMT3, and (**c**) AMMT5 samples, (**d**) pore size distribution of each specimen (SEM images were taken in 300× magnification, Scale bar: 100 µm).

SEM measurements were conducted to investigate the internal morphology of cryogels, as shown in Figure 4a–c. Cryogels showed interconnected macroporous morphology whose pore size can reach 100 µm or more. The incorporation of MMT particles into cryogels imparted roughness to the cryogel surfaces (Figure 4b,c), while smooth topology was observed in AMMT0 (Figure 4a). MMT also reduced pore size due to restriction of ice growth during the freezing process in cryogelation, also reported by Tuncaboylu et al. [40] and Koshy et al. [35]. MMT particles were uniformly distributed through matrices. At the same time, they were mostly entrapped inside pore walls. Pore size and pore size distribution were obtained via Image J analysis on SEM images, shown in Figure 4d. Average pore size for each sample was calculated as 143 ± 55 µm for AMMT0, 121 ± 33 µm for AMMT3 and 114 ± 28 µm for AMMT5 samples, respectively. In conclusion, the average pore size of alginate/MMT samples were gradually decreased with increased MMT amount due to restriction of ice growth during cryogelation method as a negative replica of the pores [40].

**Figure 5:**
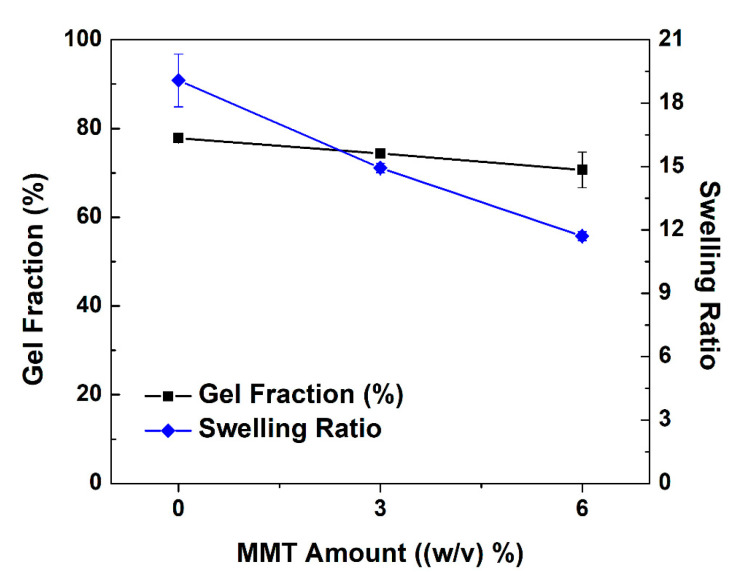
Weight swelling ratio with respect to dry state (q_w_) and gel fraction (Wg) (%) of alginate/MMT composite cryogels as a function of MMT content ((*w/v*) %). Measurements completed at least triplicate.

Gel fraction (W_g_, %) and swelling ratio (q_w_) of cryogels as a function of MMT content ((*w/v*) %) were illustrated in Figure 5. The rise in MMT amount slightly decreased the gel fraction of cryogels; however, a sharp decrease in water uptake capacity was recorded. Usually, when gel fraction increases, swelling ratio decreases based on our literature research [1]; however, this observation is mainly reported by gels prepared by ionic cross-linking; therefore, it may be logical to observe these changes in covalently cross-linked networks. The slight decrease in gel fraction could be related to the covering of inorganic particles onto cross-linking points of polymer. The swelling ratio of cryogels decreased by 50% when MMT amount was raised from 0 (*w/v*) % to 5 (*w/v*) %. This is because MMT thickened pore walls and plugged some of the pores available for water flow. The same observations were reported in nanohydroxyapatite-collagen cryogels [41] and aluminum oxide-polyacrylamide cryogels [42].

**Figure 6:**
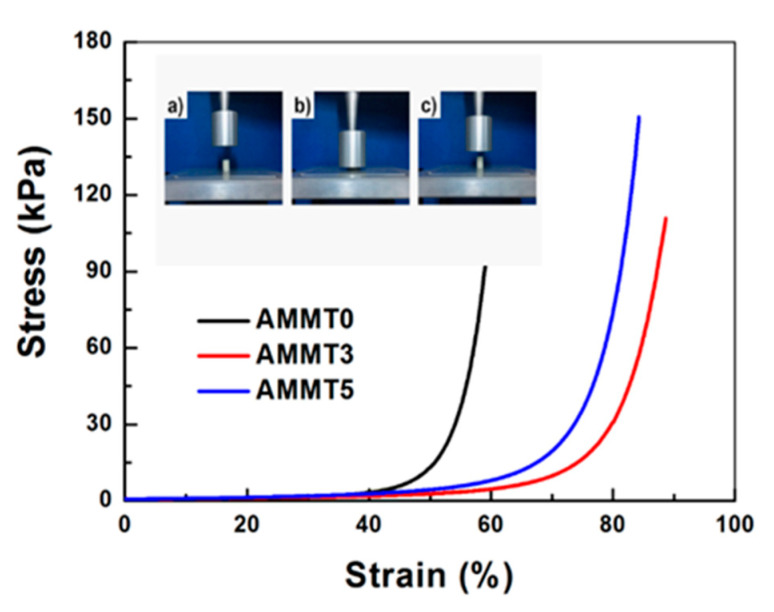
Typical stress–strain curve for AMMT0, AMMT3, and AMMT5 cryogels. (Inset: Digital images of AMMT3 (**a**) before, (**b**) during, and (**c**) after uniaxial compression test).

A uniaxial compression test was performed on alginate/MMT composite cryogels to investigate the effect of MMT amount on mechanical behavior. Figure 6 shows the stress–strain curve of each alginate/MMT cryogel. The concave upward curve for elastomeric materials was observed for all cryogels, and they were able to compress up to 80% without any breakage [43]. After the release of load in the compression test, they quickly reabsorbed water released during compression. MMT also enhanced compression stress of the cryogels, whereby their compression limit was attained to 160 kPa stress values. In cryogel systems, denser and thin pore walls yield structural support for these alginate cryogels [44]. Elastic moduli of each cryogel was calculated as 2.387 ± 0.085 kPa for AMMT0, 2.558 ± 0.111 kPa for AMMT3, and 3.697 ± 0.157 kPa for AMMT5, respectively. MMT significantly enhanced elastic moduli, about 1.5 fold, acting as a reinforcing agent. The same observations were reported by Suner et al. [45] and El-Naggar et al. [46], where they reinforced cryogels with clays. Thus, similar stress–strain curves for alginate cryogels were also reported by Kumar et al. [24], whereby they prepared agarose-alginate cryogel for biomedical applications.

### 2.2. Methylene Blue Removal

**Figure 7:**
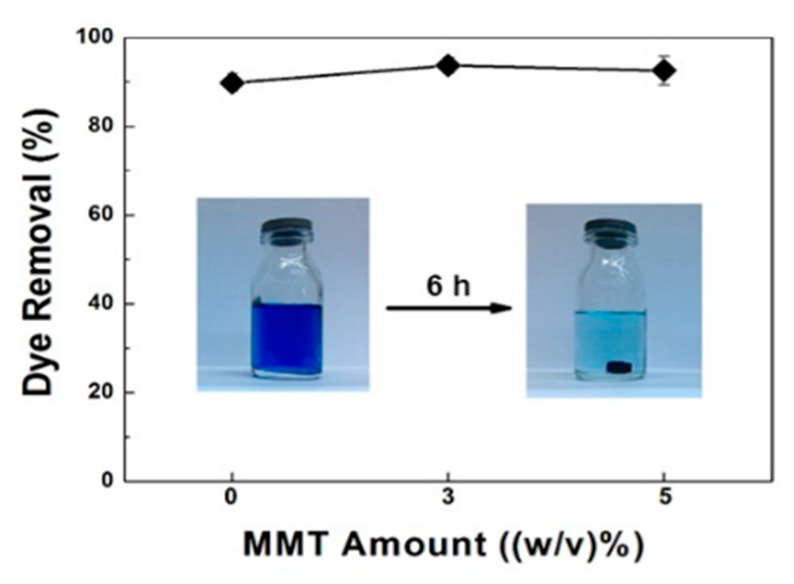
Dye removal (%) by composite cryogels with varying MMT content (inset: digital image of MB solution before (left) and after (right) 6 h contact with sample containing 3 (*w/v*) % of MMT) (initial dye concentration: 25 mg L^−1^, adsorbent dosage: 0.01 g, experiment duration: 6 h).

The impact of MMT content incorporated into cryogels on the MB removal at equilibrium is shown in Figure 7. Since all cryogels exhibited open porous structure and high surface area, they removed up to 90% of the initial MB amount. This can be explained by the cationic nature of MB and the anionic nature of alginate, whereby the gel fraction results also proved that there was still unbound COO^-^ groups for MB attachment [1]. MMT addition also enhanced dye removal by 4% due to negative charges on MMT particles [47]. When the ratio of MMT was overly increased, dye removal was slightly decreased. This could be explained by less water uptake, yielding reduced contact with MB. The decline in removal with the addition of an excess amount of clay is also reported for another adsorbent such as alumina-polyacrylamide composite cryogel [42].

**Figure 8:**
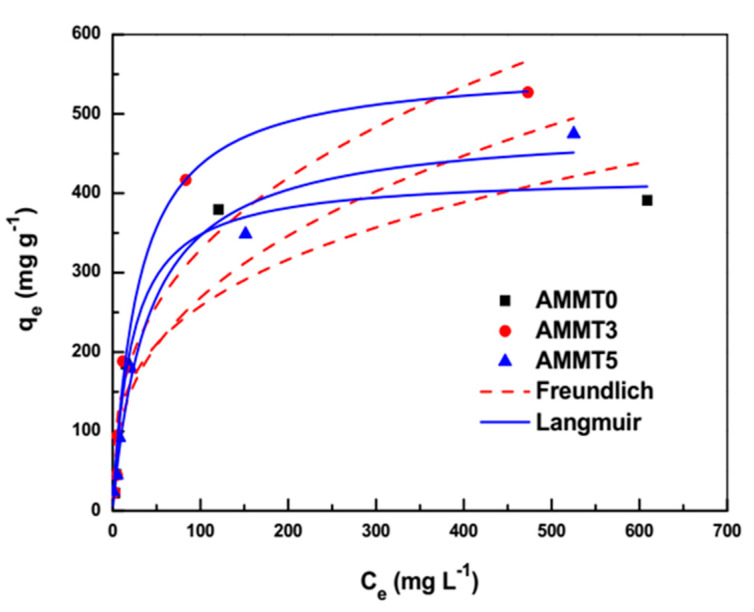
Batch adsorption data of AMMT0, AMMT3, and AMMT5 and non-linear fitting of isotherm models. Batch mode experiments were completed at MB concentrations ranging from 25 to 1000 mg L^−1^. Adsorbent dosage, MB volume, contact time, and reaction temperature were adjusted to 0.01 g, 0.01 L, 24 h, and 25 °C, respectively.

A batch adsorption study was conducted to investigate MB’s adsorption characteristics by AMMTX composite cryogels. The experimental batch adsorption data and non-linear fitting of isotherm models are illustrated in Figure 8. The highest value of the amount of dye adsorbed per adsorbent in equilibrium was observed with the AMMT3 sample independently of the initial dye concentration. Figure 8 also shows the ‘’L’’ type isotherm, whereby the ratio between dye adsorbed and dye remaining in equilibrium is a concave curve [1]. Langmuir and Freundlich’s models were utilized to analyze the relation between dye remaining in solution and dye amount adsorbed onto cryogels in equilibrium. The Langmuir model illustrates uniform and monolayer adsorption through homogeneous surfaces, while the Freundlich model explains heterogeneous adsorption where heat distribution is non-uniform [1]. The non-linear form of Langmuir (Equation (1)) and Freundlich (Equation (2)) models were described as follows:

q_e_ = (q_m_K_L_C_e_)/(1 + K_L_C_e_) (1)

q_e_ = K_F_C_e_^N^ (2)

where q_m_ is the maximum adsorption capacity or theoretical adsorption limit of sorbent (mg g^−1^) and K_L_ is Langmuir constant (L mg^−1^) [2]. K_F_ is Freundlich constant (mg g^−1^) and N is related to the nature of adsorption. Further, we calculated the feasibility of the adsorption process given by:

R_L_ = 1/(1 + K_L_C_i_) (3)

where R_L_ is the dimensionless constant, describing the feasibility of the adsorption process [1]. These parameters were calculated by fitting isotherm models onto batch experimental data and illustrated in Table 1.

Based on a high correlation coefficient (R^2^) that indicates the degree of fitting of isotherm models, the higher value obtained in the Langmuir model, indicating homogenous adsorption, where active sites for dye attachment were equally distributed all over the macroporous networks. The theoretical maximum adsorption capacity for AMMT3 was found as 559.9457 mg g^−1^, which is relatively high compared to available adsorbents such as poly(vinyl alcohol)-chitosan-alginate-MMT hydrogel beads (137.2 mg g^−1^) [8], alginate-MMT quasi-cryogel beads (181.8 mg g^−1^) [7], and alginate-clinoptilolite beads (452.25 mg g^−1^) [1], cellulose-derived carbon montmorillonite (138 mg g^−1^) [48], magnetic alginate-rice husk composite (274.9 mg g^−1^) [13]; however, relatively low compared to adsorbents such as mesoporous synthetic hectorite clay-alginate composite (785.45 mg g^−1^) [49], calcium alginate-bentonite-activated carbon beads (756.97 mg g^−1^) [4], alginate-bentonite beads (2024 mg g^−1^) [50], porous calcium alginate membranes (3506.4 mg g^−1^) [51]. R_L_ values are found to be between 1 and 0, indicating that adsorption is favorable for all cryogels.

**Figure 9:**
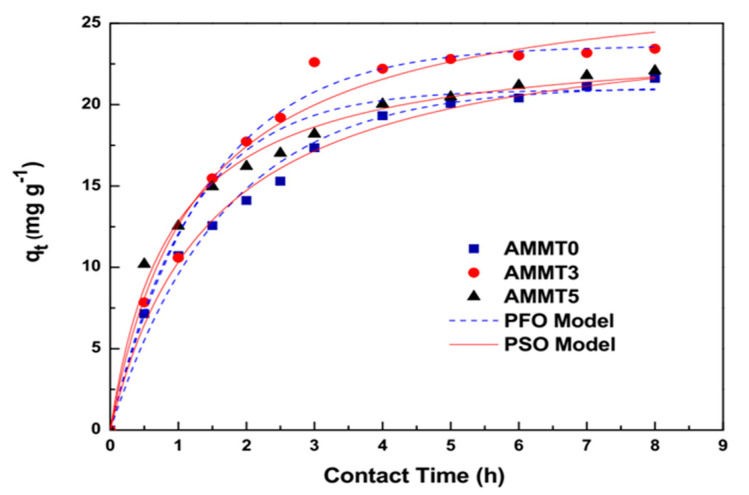
Non-linear regression of kinetic models on the MB retention by AMMT0, AMMT3, and AMMT5 samples (initial dye amount: 25 mg L^−1^, adsorbent dosage: 0.01 g, temperature: 25 °C, volume: 10 mL).

A kinetic study was completed to understand the mechanism of the adsorption process. Two kinetic models, Pseudo-First Order (PFO) and Pseudo-Second Order (PSO) models, were applied to the data, as shown in Figure 9. As can be seen, all cryogel reached their equilibrium after about 6 h of contact with the MB solution, whose adsorption capacities reached around 20 mg g^−1^. The fast removal of MB is obtained due to the open macroporosity of these cryogels, allowing the rapid flow of water and fast diffusion of dye particles [52]. However, extensive amounts of MMT hindered active sites for MB attachment resulting in reduced adsorption capacity.

PFO and PSO kinetic models were employed to characterize adsorption mechanisms of MB by alginate/MMT cryogels. PFO kinetic model favors the physisorption mechanism. The PSO kinetic model is based on the theory that the limiting step of adsorption rate covers the sharing or transfer of electrons between adsorbent and sorbent. Non-linear forms of PFO and PSO kinetic models are illustrated by Equations (4) and (5), respectively:

q_t_ = q_e_(1 + e^−k^_1_^t^) (4)

q_t_ = (k_2_q_e_^2^t)/(1 + q_e_k_2_t) (5)

where q_e_ is the adsorption capacity of cryogels in equilibrium (mg g^−1^), k_1_ is PFO rate constant (h^−1^), and k_2_ is PSO rate constant, respectively [1]. The parameters of kinetic models calculated from experimental data are shown in Table 2. Considering high R^2^ values, adsorption of MB is better described by PSO kinetic model, revealing that adsorption of MB onto cryogels has a rate-limiting step [1]. In accordance, it was also determined in previous studies where alginate-MMT quasi-cryogel beads and alginate/bentonite composite were applied for MB removal that PSO kinetic model well describes the MB adsorption [7,53].

## 3. Conclusions

Macroporous hydrogels, so-called cryogels, are sustainable materials for wastewater treatment. In the current study, we prepared alginate cryogels with a one-step cryogelation technique. Additionally, MMT was incorporated into alginate cryogels to improve mechanical strength and dye removal performance. For the cryogelation reaction, we benefitted from carbodiimide chemistry to cross-link carboxylic groups on the alginate with di-amine terminated cystamine. Unlike the calcium cross-linked cryogel-like structures of the alginate, these cryogels exhibited interconnected and open porous structures. With the one-step cryogelation technique, any shape of alginate cryogels with homogeneous cross-linking can be prepared via chemical cross-linking in the moderately frozen state. All cryogels exhibited high water uptake capacity and gel fraction values. While MMT decreased water retention capacity, it enhanced the mechanical elasticity and elastic modulus of the alginate cryogels. MMT also improved dye removal capacity against MB, which was found as 93.7%. The Langmuir model was more suitable to explain MB adsorption, whereby homogenous adsorption was observed. The maximum adsorption capacity was found as 559.74 g g^−1^, which is a relatively high value compared to other alginate/MMT adsorbents. Adsorption of MB onto alginate/MTT cryogels were better described by the Pseudo-Second Order kinetic model, revealed by the linear regression of kinetic models onto experimental data. Therefore, we can conclude that these macroporous adsorbents can potentially remove cationic dyes from wastewaters.

## 4. Materials and Methods

### 4.1. Materials

Alginic acid sodium salt from brown algae (alginate, low viscosity, Sigma, Tokyo, Japan), N-Hydroxysuccinimide (NHS, Sigma, Tokyo, Japan), 2-(N-Morpholino) ethane sulfonic acid hydrate (MES, Sigma, Tokyo, Japan), Cystamine hydrochloride (Cys, Sigma, Tokyo, Japan), N-(3-Dimethylaminopropyl)-N′-ethyl carbodiimide hydrochloride (EDC, Sigma, Tokyo, Japan), Montmorillonite K10 (MMT, Sigma, Tokyo, Japan), and Methylene blue (MB, Sigma, Tokyo, Japan) were used as received. The Milli-Q system supplied distilled water in all experiments. The molecular weight of alginate was found as 110 kDa by gel permeation chromatography analysis.

### 4.2. Preparation of Alginate/MMT Cryogels

**Figure 10:**
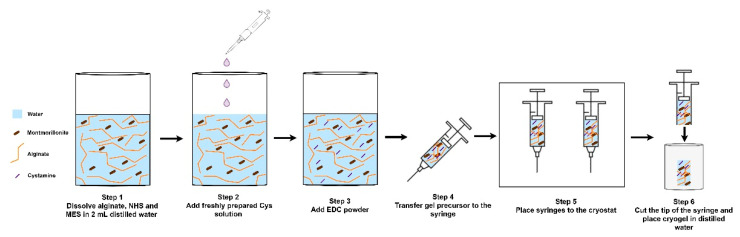
Schematic representation of the preparation of gel precursor of alginate/MMT cryogels and transfer of gel precursor solution to the cryostat.

Specific amounts of alginate, 28 mg NHS and 62 mg MES were dissolved in 2 mL of distilled water to obtain the molar ratio of COO-:NHS as 2:1, and a suitable amount of MMT was also added into this mixture and mixed for 40 min at 25 °C. Later, a fresh solution of 112 mg Cys in 100 µL distilled water was added into the solution and further mixed for 10 more minutes. A certain amount of EDC was added, mixed quickly, and transferred into disposable syringes, then replaced into cryostat at −18 °C for cryogelation of 48 h. The preparation route for alginate/MMT cryogels are shown in Figure 10. In polymeric gel precursor solution, the mole ratio of COO-:NHS:EDC was obtained as 2:1:2. After complete cross-linking, all specimens were removed from molds, washed several times with distilled water, and freeze-dried for further use. Alginate/MMT cryogels were designated as AMMTX, where X refers to the amount of MMT ((*w/v*) %) incorporated into cryogels.

### 4.3. Characterization of Alginate/MMT Cryogels

We characterized AMMTX cryogels in terms of FT-IR, SEM, gel fraction and swelling, mechanical testing, and adsorbent for MB removal.

FT-IR analysis was performed to ensure cross-linking reactions between alginate chains. IR spectra were recorded by FT-IR spectrophotometer (Shimadzu IR-Prestige 21) with Attenuated Total Reflection (ATR) unit. The spectra of dry samples were taken three times in the wavenumber ranging from 4000 to 650 cm^−1^. The spectrum was recorded three times for each specimen, whereby each spectrum was recorded after averaging 32 IR scans.

Scanning Electron Microscopy (SEM, Zeiss EVO LS 10) was used to evaluate the internal morphology of dry cryogels. Before imaging, the specimens were cut with a razor blade then sputter-coated with gold-palladium with a standard protocol (creating 5–10 nm gold-palladium layer). SEM images were taken in secondary electron mode at 20 kV.

To evaluate cross-linking efficiency of cryogels, gel fraction (W_g_, %) was calculated by:

W_g_(%) = [(M_dry_/M_0_) × 1/(0.05 × X_MMT_)] × 100 (6)

where M_0_ is the weight of samples after preparation (g), M_dry_ is the weight of dry specimens (g), and X_MMT_ is the fraction (*w/v*) of MMT in the reaction medium [19].

To determine water retention capacity, equilibrium volume and weight swelling ratios of the samples with respect to dry states were calculated. The equilibrium weight-swelling ratio concerning dry states (q_w_) of the samples was calculated as:

q_w_ = (M_sw_/M_dry_) (7)

where M_sw_ is the weight of swollen equilibrium cryogels (g) [19]. All measurements were repeated at least three times at room temperature.

A uniaxial compression test was performed with a load cell via Texture Analyzer TA.HD.Plus (Stable Micro Systems, Godalming, UK) with the computer analyzer system Texture Exponent 32 at room temperature. The compression speed was adjusted at 0.1 mm s^−1^, and tests were performed up to 80% compression of the original size of the samples in the swollen equilibrium state. The elastic modulus of each specimen was calculated by the slope of the initial linear part of the stress–strain curve [19].

### 4.4. Dye Removal

Adsorption of MB dye on the specimens was conducted by batch procedure and kinetic study with a magnetic stirring at a speed of 200 rpm at 25 °C. In each experiment, 0.01 gr dry adsorbent was soaked into 10 mL of MB solution. MB concentration in remaining solutes was measured using UV-Vis Spectrophotometer (UV-1700 PharmaSpec, Schimadzu, Kyoto, Japan) at 665 nm [1]. Each experiment was conducted three times.

For the batch procedure, MB solutions were prepared with varying concentrations (25 to 1000 mg L^−1^), and the experiment was conducted for 24 h. The amount of dye adsorbed in equilibrium (q_e_, mg g^−1^) was calculated by Equation (8):

q_e_ = (C_i_ − C_e_) × V/M (8)

C_i_ and C_e_ refer to the dye concentration in aqueous solution before (mg L^−1^) and after (mg L^−1^) adsorption. V is the volume of the aqueous medium (L) and M is the dosage of dry adsorbent (g) [2].

A 25 mg L^−1^ MB solution was employed for kinetic study, and an experiment was conducted for 8 h. The amount of dye adsorbed onto cryogels per time (qt, mg g^−1^) was calculated by Equation (9) given as:

qt = (C_i_ − Ct) × V/M (9)

where Ct refers to the concentration of the MB dye remaining in solution at time t (mg L^−1^) [2].

Dye removal per sorbent (%) was also calculated using equations as follows:

Dye removal (%) = (C_i_ − C_e_)/C_i_ × 100 (10)

where C_i_ and C_e_ are the same as Equation (8) [1].

## Figures and Tables

**Table 1:** Langmuir and Freundlich isotherm constants for MB adsorption onto composite cryogels.

Cryogel	Langmuir Isotherm	Freundlich Isotherm
AMMT0	q_m_ (mg g^−1^), 422.880	K_F_, (mg g^−1^), 65.958
	K_L_ (L mg^−1^), 0.046	N, 0.296
	R^2^, 0.980	R^2^, 0.790
	R_L_, 0.460	
AMMT3	q_m_ (mg g^−1^), 559.950	K_F_, (mg g^−1^), 65.583
	K_L_ (L mg^−1^), 0.035	N, 0.3502
	R^2^, 0.981	R^2^, 0.870
	R_L_, 0.530	
AMMT5	q_m_ (mg g^−1^), 462.770	K_F_, (mg g^−1^), 51.142
	K_L_ (L mg^−1^), 0.028	N, 0.356
	R^2^, 0.990	R^2^, 0.950
	R_L_, 0.590	

**Table 2:** PFO and PSO kinetic parameters for MB adsorption onto AMMT0, AMMT3, and AMMT5 samples at the initial dye concentration of 25 mg L^−1^.

Cryogel	PFO Kinetic Model	PSO Kinetic Model
AMMT0	q_e,calc_ (mg g^−1^), 21.13	q_e,calc_ (mg g^−1^), 25.62
	k_1_ (min^−1^), 0.010	k_2_ (min^−1^), 0.004
	R^2^, 0.980	R^2^, 0.990
AMMT3	q_e,calc_ (mg g^−1^), 23.62	q_e,calc_ (mg g^−1^), 28.26
	k_1_ (min^−1^), 0.017	k_2_ (min^−1^), 0.005
	R^2^, 0.980	R^2^, 0.980
AMMT5	q_e,calc_ (mg g^−1^), 20.93	q_e,calc_ (mg g^−1^), 24.07
	k_1_ (min^−1^), 0.014	k_2_ (min^−1^), 0.008
	R^2^, 0.960	R^2^, 0.990
